# Role of Lifetime Body Mass Index in the Association Between Age at Puberty and Adult Lipids: Findings From Men and Women in a British Birth Cohort

**DOI:** 10.1016/j.annepidem.2010.05.015

**Published:** 2010-09

**Authors:** Mary B. Pierce, Diana Kuh, Rebecca Hardy

**Affiliations:** MRC Unit for Lifelong Health and Ageing, London, United Kingdom

**Keywords:** Body Mass Index, Lipids, Longitudinal Study, Puberty, CVD, cardiovascular disease, BMI, body mass index, LDL, low-density lipoprotein, HDL, high-density lipoprotein, 95% CI, 95% confidence interval

## Abstract

**Purpose:**

Why early puberty is associated with worse cardiovascular outcomes is unknown. The relationship between puberty and lipids is unclear. Our aim was to assess whether age at puberty was associated with triglyceride and total low-density lipoprotein and high-density lipoprotein cholesterol at age 53 years.

**Methods:**

Participants in a national birth cohort were examined at 15 years, when pubertal stage for boys was assessed and age at menarche reported by the girls' mothers. At 53 years, 3035 were interviewed in their homes by research nurses, where blood was taken.

**Results:**

There was a significant inverse relationship in women but not men between age at puberty (in years) and triglycerides (regression coefficient −0.2.9, 95% confidence interval −5.5, to −0.04, *p* = .02), age at puberty, and age at puberty and adult body mass index (BMI; *p* < .001). Relationships between puberty and lipids were completely explained by BMI or waist circumference at 53 years.

**Conclusions:**

In both sexes earlier maturation was associated with greater BMI and waist circumference in later life, which resulted in greater triglycerides and cholesterol in women. We suggest that intervention after puberty to help avoid obesity in early maturing women may improve their later cardiovascular health.

## Introduction

Better understanding of the relationships between age at puberty and risk factors for cardiovascular disease (CVD) may lead to improved management of CVD risk. An inverse relationship between age at menarche and ischemic heart disease has been observed in a number of studies (1–3), but the reason for this relationship remains unclear.

Remsberg et al. (4) demonstrated that girls who have an early puberty (<12 years) have worse cardiovascular risk factors throughout adolescence than girls with later menarche. Other researchers (5–7) have demonstrated that cardiovascular risk factors, including lipids, track within subjects through adolescence and into early adulthood. If this tracking persists into later life, early menarche would be expected to be associated with worse cardiovascular risk factors at older ages. The authors of many longitudinal studies (8–15) have found an inverse relationship between age at menarche and adult body mass index (BMI). The relationship of age at menarche with lipids and lipoproteins has been less well studied, and results are contradictory (13, 14, 16, 17).

Furthermore, it is unclear whether any effect of age at menarche on lipids is independent of body size. The authors of a Finnish study (14) concluded that age at menarche is simply a risk marker, with high BMI in childhood leading to earlier menarche as well as greater adult BMI and associated greater CVD risk. However, in the analyses, what they called “childhood” BMI was in fact BMI measured around puberty. Pierce and Leon (18) previously found that true childhood BMI measured between 4 and 6 years did not explain the relationship between age of menarche and adult BMI.

Little research has considered the association between age at puberty and CVD risk in men because it is more difficult to assess puberty in boys. In the Medical Research Council National Survey of Health and Development, we have previously shown that men, but not women, who experienced early puberty had greater systolic and diastolic blood pressure at 53 years than those with later puberty and that people with early onset of puberty have a greater BMI in later life (15). Building on this previous work, by using the same cohort, we consider whether age at puberty in both sexes was associated with triglycerides and total cholesterol, low-density lipoprotein (LDL), and high-density lipoprotein (HDL) levels at age 53 years. We assessed whether any association was explained by current body size. Finally, we investigated whether any association, as well as the previously observed association between early puberty and high adult BMI, was accounted for by childhood (age 7 years) or pubertal (age 11 years) body size.

## Methods

The Medical Research Council National Survey of Health and Development is a birth cohort study of a sample of 2547 women and 2815 men born in 1 week in March 1946 (19, 20) in England, Scotland, and Wales. The cohort has been followed up 21 times since birth. A total of 3035 cohort members provided information at age 53 years, a response rate of 83% for the 3673 for whom contact was attempted. The majority were interviewed and examined in their own homes by trained research nurses, with others completing a postal questionnaire (n = 46). Contact was not attempted for 1976 (37%) subjects who had previously refused to take part (12%), were living abroad (11%), were untraced since their last contact at 43 years (5%), or had already died (9%). The responding sample at 53 years was in most respects representative of the national population of the same age, but tended to under-represent the most disadvantaged groups compared with census data (21). The data collection received Multicentre Research Ethics Committee approval, and informed consent was given by respondents to each set of questions and measures.

Total cholesterol, HDL, and LDL levels were obtained from nonfasting venous samples taken by the nurses at home visits when participants were 53 years of age. The analytes were all measured in 2000 at the Royal Victoria Hospital, Newcastle, England. Total cholesterol was measured by enzymatic CHOD-PAP (Randox Laboratories Ltd., Antrim, United Kingdom). Precipitation for measurement of HDL cholesterol was performed with the use of phosphotungstic acid-Mg^2+^; triglycerides were measured using a glycerol/kinase POD-linked reaction of glycerol liberated enzymatically from triglycerides. All of these measurements were made with a Bayer DAX-72. LDL cholesterol was calculated by use of the Friedwald formula; LDL cholesterol= total cholesterol − HDL cholesterol − 0.45 × triglycerides. Height and weight were measured at the home visits according to standardized protocols. BMI (weight in kg/[height in meters ]^2^) was calculated.

At age 15 years of age, study members underwent medical examinations and interviews by school doctors. The physical examinations, which predate Tanner staging, included assessments of the visibility of pigmented pubic hair (none, sparse, profuse), the visibility of axillary hair (no, yes) and, for boys only, the development of genitalia (infantile, early, advanced), and whether the voice had broken (no, starting, completely broken). On the basis of these four indicators, boys were classified as infantile (infantile genitalia or early adolescent genitalia, no pubic or axillary hair and voice not broken), early puberty (early development of genitalia some pubic or axillary hair or voice starting to break), advanced puberty (advanced development of genitalia but at least one other marker not fully mature), and fully mature (advanced development of genitalia, profuse pubic and axillary hair and voice broken). Age at menarche for girls was obtained from mothers' reports at the examination. Because 188 girls had not reached menarche by the date of the examination, the exact age at menarche obtained from cohort members' self-reports when they were aged 48 years was used where available (n = 94). At ages 7 and 11 years the children's heights and weights (in underclothes) were measured by school nurses and doctors.

During the interview at 53 years, the research nurses recorded information on participants' current medications. This information was coded according to the British National Formulary, and participants who were taking British National Formulary classified lipid-lowering drugs were distinguished from those not taking those drugs.

### Statistical Analysis

The values for triglycerides were log transformed because they were not normally distributed. All analyses were carried out separately for men and women. To account for subjects on lipid-lowering medication (n = 57) at the time the lipids were measured, censored Normal regression models were used to examine the relationship between age at menarche in girls or pubertal stage at age 15 years in boys and total cholesterol, HDL and LDL cholesterol, and triglycerides. These models censor the outcome of subjects on medication at the observed value, so that the true lipid value is assumed to be at least as high as that on treatment (22). For HDL cholesterol, where treatment has little effect on the level, we used standard normal regression models.

Age at menarche was fitted as a continuous variable and tests for deviation from linearity were carried out. Regression (beta) coefficients were obtained from these models representing the mean change in lipid per year increase in age at menarche. To assess whether results were influenced by the greater exclusion of those with late menarche, we also analyzed age at menarche as a categorical variable. We defined four groups with all 188 who had not reached menarche at the examination included in the latest menarche group. For men, a test for trend was carried out across the four pubertal stage groups. Where there was no deviation from a linear trend further models were fitted with pubertal stage as a continuous variable. To assess whether any observed associations were explained by current body size, we adjusted the initial models for BMI, and then separately for height at 53 years, and then separately for waist circumference (a better measure of intra-abdominal fat than BMI) at 53 years (23).

To assess whether any relationships between age at puberty and lipids were influenced by childhood adiposity, we adjusted the initial models for BMI at age 7 years (childhood BMI) and then separately for BMI measured at 11 years (pubertal BMI). Finally, we carried out a similar analysis with BMI at 53 years as the outcome. Analyses were carried out in SPSS 14 (SPSS, Chicago, IL), and STATA 10 (College Station, TX).

## Results

A total of 1047 men and 999 women of the 3035 interviewed at age 53 years who had valid measures of pubertal stage (for men), age at menarche (for women) BMI, waist circumference and any one of the lipid measures (triglycerides, total and LDL and HDL cholesterol; Table 1) were eligible for analysis. For boys, 24% were in the early puberty group and 10% in the late group. For girls, the mean age at menarche was 12.7 years.

Those who were not interviewed at 53 years were more likely to be from a manual social class in childhood (women, *p* = .01) and adulthood (men, *p* < .001) than those interviewed, but there was no significant difference in age at puberty between the two groups. Between those included in regression analyses and those who were excluded because of missing covariate data there were some statistically significant, but clinically very small differences in the women, with those excluded having higher mean cholesterol and triglyceride levels (<0.1 mmol/L).

In women, there was a significant linear inverse relationship between age at menarche and triglycerides (*p* = .02; Table 2). A similar, although weaker (*p* = .06), relationship was observed between age at menarche and total cholesterol (Table 2). There was no evidence of a relationship with LDL or HDL cholesterol. The associations with total cholesterol and, in particular, with triglycerides were attenuated on adjusting for current BMI or current waist circumference and were no longer significant. The associations were also slightly attenuated by adjusting for current height. In men, there were no significant relationships between timing of puberty and total, LDL or HDL cholesterol, or triglycerides (Table 3). Adjustment for BMI or height had little impact on these findings.

The unadjusted relationship between age at menarche and triglycerides was slightly weaker in the sub-sample with complete information on early BMI (Table 4) because of reduced power, but the regression coefficient was little altered. Adjustment for BMI at age 11 years attenuated the relationship between age at menarche and triglycerides from −2.6% (95% confidence interval [95% CI], −5.4 to 0.5) to −1.8% (95% CI, −4.8 to 1.2). In contrast, the association was unchanged when adjusting for BMI at age 7 years. The relationship between age of menarche in girls and adult cholesterol was unaltered by adjustment for BMI measured at either age 7 years or 11 years (Table 4). The unadjusted association in the subsample with complete data on BMI (Table 4) was weaker than that in the full sample (Table 2), but the regression coefficient remained unchanged. Among the boys, the non significant associations remained unchanged after adjusting for either BMI measure (Table 4).

In both sexes, earlier onset of puberty was significantly associated with greater BMI at 53 years (Table 4). In girls, the relationship between age at menarche and adult BMI was reduced after adjustment for childhood BMI measured at age 7 years, although the association remained significant (−0.52 kg/m^2^; 95% CI, −0.79 to −0.25) per year increase in age at menarche (*p* < .01; Table 4). Adjustment for pubertal BMI measured at 11 years attenuated the relationship to a greater extent, and the relationship was no longer significant (*p* = .09). Similar results were seen in boys (Table 4).

Analyses in which we used a categorical age at menarche variable produced results (not shown) that were consistent, although weaker because of reduced power, with analyses presented with age at menarche as a continuous variable.

## Discussion

Our findings suggest that age of onset of puberty has little independent effect on lipid or lipoprotein levels in late middle age in either sex. Women with an earlier menarche had greater triglycerides and cholesterol than those maturing later. This finding was largely a result of those reaching puberty earlier having a greater BMI and waist circumference in later life. There were no such relationships in men.

### Advantages and Limitations

This is one of very few studies examining the relationship between age of menarche and lipids. Most previous studies (14) were either cross-sectional, relying on recalled age of menarche (13, 16), which is subject to recall bias (24), or the authors measured the risk factor outcomes in late adolescence or early adult life (4, 16). None of the other studies have repeated measurements of weight and height in the same children in early and later childhood. To our knowledge, no other researchers have investigated age at puberty and lipids in men. Our study assessed age at menarche during adolescence and physically examined the boys for pubertal stage at age 15 years. We do not have an exact age at menarche for girls who had not reached menarche by age 15 years. Exclusion of a greater proportion of the later maturing girls, and imputation of recalled values for some using recalled information, may result in bias in our study. However, the similar, although weaker, relationships found using menarche as a categorical variable with those who had not reached menarche by 15 years included in the latest maturing group, suggests little evidence of bias.

The responders at 53 years tended to underrepresent the most disadvantaged compared with census data but were in most respects representative of a national sample of a similar age (21). Loss to follow-up and missing data are inevitable in long-running cohorts (21). There is no reason to suspect that any differences would have had a substantial impact on our findings. Importantly, there was no difference in the distribution of age at puberty between those excluded from the analysis and those included. Because lipids were not measured in childhood, it was not possible to address whether our results are affected by tracking of lipids from childhood into adulthood.

### Comparisons With Other Studies

Two recent cross-sectional studies assessing the relationship between age at menarche and lipids/lipoproteins in midlife were from Chinese investigators (13, 16). Our finding of an inverse relationship between age at menarche and triglycerides that is attenuated by adult BMI and adult waist circumference is in similar to the findings of both of these studies. Feng et al. (13) found that adjustment for body size substantially attenuated the relationship between age at menarche and triglycerides, and Heys et al. (16) reported that adjustment for waist circumference attenuated but did not completely explain the relationship. The Fels longitudinal study (4), which followed girls between age 8 and 21 years, also suggested that girls with early menarche had less favorable changes in triglycerides that were mediated by body composition. However, this study did not distinguish between pre- and postmenarcheal risk factor measures.

Our finding of an unadjusted relationship between age of menarche and total cholesterol is in contrast with previous longitudinal studies that have found no association (4, 14, 17), but none have follow-up into late middle age. The Fels longitudinal study (4) found no relationship between age at menarche and changes in cholesterol levels during adolescence. In the Bogalusa Heart Study (17), investigators followed girls up to the third decade, and a birth cohort study from Finland (14) followed approximately 700 women until they reached 30 to 39 years of age. Only one (13) of the two cross-sectional Chinese studies, which have measures of lipids in later life, addressed the relationship between age of menarche and total cholesterol and also found no relationship.

Feng et al. (13) found a significant relationship with HDL cholesterol because of the large sample size, but the reported effect was very small in clinical terms (0.007 mmol per year). Our study did not have the statistical power to detect such a small and clinically unimportant association. Our effect size was 0.01 mmol per year. The other Chinese study, like ours, did not show an association between age at menarche and HDL cholesterol (16).

Since 1972 many longitudinal studies have reported a negative relationship between age of menarche and adult weight for height (8–14, 18, 25, 26). Our finding that this association was largely explained by BMI measured at 11 years is in agreement with a Finnish study (6), the 1958 birth cohort (10), and the Bogalusa Heart Study (26). We, however, also find that adjustment for BMI earlier in childhood (at age 7 years) explains much less of the association. We argue, as we did previously (18), that the effect of adjustment for childhood BMI differs depending on the age at which BMI is assessed, because the determinants of variation in BMI in early childhood and later childhood are different. If assessed at 7 years or earlier (18), childhood BMI is largely unaffected by pubertal changes. However, if childhood BMI is measured later in childhood, that is, at 9 years or older as in the previous three studies (6, 10, 26), it reflects to a greater degree the changes in growth and body composition associated with the process of early pubertal development and sexual maturation. Our findings therefore suggest that it is pubertal BMI, rather than BMI in childhood, that tracks to adulthood and results in worse lipid levels in women. Although preventing obesity in childhood is laudable for other reasons, it may not deal with the additional problem related to adult obesity faced by young people experiencing early menarche. We suggest that if investigators wish to examine the effect of childhood BMI, they should be using early measurements of BMI.

Our study suggests that the associations with lipid levels in middle age are driven by adult body size. The mechanism for the association between age at menarche and later BMI is unclear. What is known is that girls who are going to have an earlier puberty are taller and fatter in late childhood, and, although there may be a minimum weight for the onset of puberty (27), this is not causal (28). Similarly, it is now thought that leptin levels are permissive rather than causal in the timing of menarche (29). Girls who have early puberty grow more rapidly in early life than those who will have later puberty and after their epiphyses fuse they appear to continue this growth by laying down fat. There is some cross-sectional evidence that girls and boys who have discordant sexual development as assessed by Tanner stages (ie, breast or genital development proceeding faster than pubic hair development) are heavier with a larger BMI than other adolescents, but it is not known whether this persists into later life (30). It has also been suggested that early-maturing girls are less likely to persist with sport in their teens (31), and that this contributes to their adiposity.

In our study the effect of age at puberty on lipids is different in the two sexes. Our findings suggest that age at puberty has little effect on lipids in men, despite early puberty being associated with high adult BMI in men as well as women. It is unclear why this greater BMI should result in poorer lipid levels in women but not men. However, our marker of puberty in boys may be measured with greater error than age at menarche in girls. Our study may have lacked the power to detect the difference between groups in men. Although no trends across pubertal growth were observed, estimates and CIs suggest that it remains possible that the later maturing boys had lower triglycerides and total cholesterol at age 53 years. Our previous findings, in contrast, suggest a stronger impact of age at puberty on BP in men than women (15). Those men who reach puberty late have lower blood pressure in middle age than others, which was not accounted for by adult body size.

In conclusion, we did not find any independent effect of age at puberty on lipids in late middle age. In both sexes earlier maturation was associated with a greater BMI in later life, and this greater BMI resulted in greater triglycerides and possibly greater cholesterol in women. We also found that the association between early puberty and high BMI in middle age in both sexes was not explained by childhood BMI but was explained by pubertal BMI. Avoiding obesity in early maturing young people may thus be important for their later cardiovascular health.

## Figures and Tables

**Table 1:** Means (SD) for continuous measures and percentages for categorical variables by sex

	Men		Women	
	n	Mean (SD)	n	Mean (SD)
Age at puberty group (boys status at 15 years)	1051	−	−	−
1 latest (%)	105 (9.99)	−	−	−
2	368 (35.14)	−	−	−
3	326 (31.01)	−	−	−
4 earliest	248 (23.60)			
Age at menarche, years	−	−	999	12.71 (1.25)
Measurements at 53 years				
Cholesterol, mmol/L	1051	6.05 (1.07)	999	6.10 (1.10)
LDL cholesterol, mmol/L	932	3.57 (0.95)	967	3.49 (1.00)
HDL cholesterol, mmol/L	940	1.51 (0.56)	968	1.83 (0.48)
Triglycerides, mmol/L	1048	2.48 (1.76)	999	1.76 (1.13)
BMI, kg/m^2^	1047	27.50 (3.88)	999	27.44 (5.36)
Waist circumference, cm	1047	97.97 (10.46)	999	85.68 (12.57)
Height, cm	1047	174.66 (6.41)	999	161.69 (6.00)

BMI = body mass index; HDL = high-density lipoprotein; LDL = low-density lipoprotein.

**Table 2:** Regression coefficients and 95% confidence intervals for the association between reported age at menarche (fitted as a continuous variable in months) and lipids at 53 years in women, unadjusted and adjusted for body mass index (BMI), waist circumference at 53 years, and height at 53 years

	Unadjusted		Adjusted for BMI at 53 years		Adjusted for waist at 53 years		Adjusted for height at 53 years	
Outcome	Regression coefficient (95% CI)	*p*	Regression coefficient (95% CI)	*p*	Regression coefficient (95% CI)	*p*	Regression coefficient (95% CI)	*p*
Triglycerides, %^a^ n = 997	−2.97 (−5.56, −0.39)	.02	0.27 (−2.18, 2.72)	.82	−0.82 (−3.12, 1.51)	.49	−2.40 (−4.97, 0.20)	.07
Total cholesterol, mmol/L n = 997	−0.05 (−0.11, 0.00)	.06	−0.03 (−0.08, 0.02)	.34	−0.03 (−09, 0.02)	.21	−0.04 (−0.10, 0.01)	.13
LDL cholesterol, mmol/L n = 965	−0.03 (−0.08, 0.02)	.21	−0.01 (−0.06, 0.04)	.78	−0.02 (−0.07, 0.03)	.50	−0.02 (−0.08, 0.03)	.35
HDL cholesterol, mmol/L n = 967	0.01 (−0.01, 0.04)	.29	0.10 (−0.03, 0.13)	.39	0.00 (−0.02, 0.02)	.99	0.01 (−0.01, 0.04)	.38

a Outcome transformed using 100 × log_e_ so that regression coefficient represents percent change in outcome per year increase in age at menarche.

**Table 3:** Regression coefficients and 95% confidence intervals for the association between puberty group and lipids at 53 years in men

	Pubertal group at age 15 years				
	Advanced puberty	Early puberty	Infantile		
Outcome	Reg. Coeff (95% CI)	Reg. Coeff (95% CI)	Reg. Coeff (95% CI)	*p* across groups	*p* for trend
Triglycerides, %^a^ n = 1048	−6.61 (−16.58, 3.36)	−2.91 (−12.63, 6.81)	−6.82 (−20.52, 6.87)	.56	.48
Total cholesterol, n = 1051	−0.15 (−0.33, 0.03)	−0.10 (−0.27, 0.08)	−0.21 (−0.45, 0.04)	.28	.17
LDL cholesterol, n = 932	−0.04 (−0.21, 0.12)	0.01 (−0.17, 0.15)	−0.16 (−0.39, 0.07)	.53	.38
HDL, mmol/L n = 940	−0.03 (−0.13, 0.07)	0.04 (−0.05, 0.14)	0.03 (−0.07, 0.14)	.44	.29

Unadjusted regression coefficients for each later puberty group compared with baseline group (fully mature at 15 years). 95% CI = 95% confidence interval; HDL = high-density lipoprotein; LDL = low-density lipoprotein.

**Table 4:** The effect of adjustment for BMI at 7 years, and BMI at 11 years, on relationship between onset of puberty and cholesterol, and lipids and BMI at age 53 years

	Unadjusted		Adjusted for BMI age 7 years		Adjusted for BMI aged 11 years	
Outcome	Regression coefficient^a^(95% CI)	*p*	Regression coefficient^a^(95% CI)	*p*	Regression coefficient^a^(95% CI)	*p*
Women						
Triglycerides (%)^b^	−2.61 (−5.41, 0.50)	.07	−2.60 (−5.4, 0.30)	.08	−1.83 (−4.84, 1.21)	.24
Cholesterol, mmol/L	−0.05 (−0.11, 0.02)	.14	−0.05 (−0.11, 0.01)	.11	−0.05 (−0.11, 0.02)	.15
LDL, mmol/L	−0.03 (−0.09, 0.02)	.29	−0.04 (−0.10, 0.02)	.19	−0.03 (−0.09, 0.02)	.25
HDL, mmol/L	0.02 (−0.01, 0.04)	.24	0.01 (−0.01, 0.04)	.33	−0.01 (−0.02, 0.04)	.44
BMI, kg/m^2^	−0.75 (−1.00, –0.48)	<.001	−0.52 (−0.79, –0.25)	<0.001	−0.23 (−0.50, 0.04)	.09
Men						
Triglycerides, %^b^	−0.60 (−4.7, 3.5)	.77	−0.70 (−3.8, 2.3)	.64	−0.50 (−4.6, 3.7)	.83
Cholesterol, mmol/L	−0.01 (−0.08, 0.06)	.82	−0.02 (−0.09, 0.06)	.66	−0.02 (−0.09, 0.05)	.63
LDL, mmol/L	0.03 (−0.04, 0.10)	.41	0.03 (−0.04, 0.10)	.39	0.02 (−0.05, 0.09)	.58
HDL, mmol/L	−0.01 (−0.04, 0.02)	.56	−0.01 (−0.04, 0.02)	.44	−0.01 (−0.05, 0.02)	.38
BMI, kg/m^2^	−0.43 (−0.69, –0.18)	<.001	−0.34 (−0.59, –0.09)	.01	−0.22 (−0.46, 0.03)	.08

BMI = body mass index; HDL = high-density lipoprotein; LDL = low-density lipoprotein.

a Regression coefficients represent mean change per year increase in age at menarche in women and mean change per group increase in men.

b Outcomes transformed using 100 × log_e_ so that regression coefficient represents percent change in outcome for each unit increase in age at puberty.
